# Multistage sleep recordings: a missing piece in the puzzle of human interictal single-neuron dynamics

**DOI:** 10.1093/braincomms/fcag273

**Published:** 2026-07-20

**Authors:** Jonathan Curot

**Affiliations:** Univ Toulouse, CNRS, CerCo, Toulouse, France; Univ Toulouse, CHU de Toulouse, Neuroscience Division, Brain Electrophysiology, Epilepsy and Sleep Unit, Toulouse, France

## Abstract

This scientific commentary refers to ‘Sleep increases firing rate modulation during interictal epileptiform discharges in mesial temporal structures’, by Whitmarsh *et al*. (https://doi.org/10.1093/braincomms/fcag130).

Since the earliest electroencephalographic observations demonstrating the facilitation of epileptic activity during non-rapid eye movement (NREM) sleep, it has become increasingly clear that sleep is not merely a permissive state for epilepsy, but an active modulator of pathological neuronal synchronisation.^1^ Yet despite extensive work using scalp and intracranial local field potentials (LFPs), relatively little is known about how sleep shapes the behaviour of individual neurons during interictal epileptiform discharges (IEDs) in humans.

In this context, the recent study by Whitmarsh *et al*.^2^ in *Brain Communications* provides an important contribution by combining prolonged macro- and microelectrode intracerebral recordings across several nights with automated detection of thousands of IEDs and detailed polysomnographic staging. This approach allowed the authors to characterise neuronal dynamics across vigilance states in patients with mesial temporal epilepsy. Such datasets remain rare in human intracranial electrophysiology, particularly when microelectrode recordings are maintained over natural sleep–wake cycles. This setting remains technically challenging because of noise levels, recording stability, and the complexity of simultaneous multimodal acquisitions.

Their main conclusions extend previous observations regarding the strong influence of sleep on interictal activity. Both the rate and amplitude of IEDs progressively increased with deeper stages of NREM sleep, reinforcing the notion that thalamocortical synchrony facilitates epileptic activity.^3^ The authors further demonstrate a frequent increase in single-unit and multi-unit firing during the spike component of IEDs, followed by a pronounced decrease during the associated slow wave. Importantly, these modulations became progressively stronger with deeper NREM sleep stages. In parallel, outside epileptic events, baseline neuronal firing rates, bursting activity, and firing regularity all decreased with increasing sleep depth.

Taken together, these observations provide a compelling picture of how physiological sleep oscillations may reshape epileptic neuronal dynamics. NREM sleep appears not only to facilitate IED occurrence, but also to amplify the contrast between transient pathological synchronisation during the spike and neuronal suppression during the subsequent slow wave. Such findings resonate with broader concepts of cortical bistability during slow-wave sleep, characterised by alternating UP and DOWN states.^1,3^

However, this dominant pattern should not obscure the marked heterogeneity of neuronal dynamics during IEDs. A debate that has shaped the single-unit epilepsy literature for more than four decades concerns whether IEDs truly represent hypersynchronous neuronal events^4^ or instead reflect sparse and heterogeneous microcircuit dynamics. While the current results may initially appear compatible with the classical hypersynchrony framework, Whitmarsh *et al*.^2^ reported that 75.9% of ‘responsive’ single units showed either increased firing, decreased firing, or both, along with substantial variability within and across patients.

A recurring lesson from human single-unit studies is that IEDs rarely recruit all nearby neurons uniformly. In Keller *et al*.’s 2010 study,^5^ only 48% of recorded units showed firing-rate modulation within 500 ms of the IED. Within this wakefulness dataset, interictal activity was ‘not a simple paroxysm of hypersynchronous excitatory activity,’ but rather the result of interactions among multiple neuronal populations embedded within complex local networks. Predischarge neuronal changes, occurring several hundred milliseconds before the visible IED, were mainly observed within the seizure-onset zone, suggesting that the LFP event represents only the surface manifestation of broader and temporally distributed neuronal processes.

Similarly, in 2013 Alvarado-Rojas *et al*.^6^ reported that approximately 30% of units increased and 40% decreased firing around IEDs, while only a very small subset discharged synchronously during the spike itself. Nearly 30% of units showed firing-rate changes preceding the IED by several hundred milliseconds, supporting the idea that synchronisation may emerge progressively through sparse and temporally distributed neuronal assemblies rather than abrupt simultaneous recruitment. Even earlier recordings already pointed toward a weak and variable relationship between unit activity and surface IEDs.^7^

Viewed in light of these previous findings, the dominant neuronal profile observed during IEDs should not give the impression of fully homogeneous firing-rate modulation. The authors explain the discrepancy with earlier reports through several methodological and clinical arguments, including automated large-scale IED detection, prolonged recordings, temporal cluster analyses, and restriction to mesial temporal structures. However, the absence of cross-correlation analyses between LFPs and single-unit activity limits our ability to interpret the temporal organisation of neuronal recruitment during IEDs.

Indeed, spike-LFP cross-correlograms are critical for determining whether neuronal firing is tightly time-locked to the epileptic discharge, progressively recruited before the visible spike, or suppressed during subsequent inhibitory phases.^8^ Such analyses may help distinguish sharply synchronised neuronal recruitment from temporally dispersed responses and clarify whether the increased firing rates observed during NREM sleep reflect stronger temporal locking to IEDs rather than a simple increase in firing probability.

Beyond these methodological aspects, an important physiological question emerges: could sleep itself partially homogenise neuronal responses to epileptic discharges?

Most previous single-unit studies were performed primarily during wakefulness or without systematic circadian and sleep-stage distinctions. By contrast, Whitmarsh *et al*.^2^ demonstrate that neuronal modulation strengthens progressively during deeper stages of NREM sleep. Such findings raise the possibility that sleep-related oscillatory states constrain neuronal activity into more coherent synchronisation regimes, as previously suggested in the mesial temporal lobe.^1^ NREM sleep is physiologically characterised by large-scale synchronisation through slow oscillations, spindles, and hippocampal sharp-wave ripples.^3^ These oscillatory dynamics may reduce the intrinsic variability of epileptic neuronal recruitment, making IED-related firing patterns appear more homogeneous than during wakefulness.

Importantly, cross-correlogram approaches could have provided further insight into this issue by determining whether neuronal firing becomes more tightly time-locked to IEDs across sleep stages, whether pre-discharge synchronisation becomes more temporally constrained during NREM sleep, or whether sleep primarily amplifies already existing neuronal couplings. Such analyses may ultimately help disentangle true hypersynchronous recruitment from the state-dependent temporal organisation of sparse neuronal assemblies.

At the same time, growing evidence argues against the idea of a unitary ‘IED phenomenon’ at the neuronal level and instead supports a taxonomy of epileptiform discharges with distinct pathophysiological significance. Not all IEDs are physiologically equivalent, a notion that is further supported by the marked variability in IED morphology across patients including in the Whitmarsh *et al*.^2^ dataset. For instance, high-frequency oscillation (HFO)-associated IEDs possess distinct neuronal signatures compared with non-HFO IEDs.^8,9^ HFO-IEDs are characterised by pronounced pre-peak firing increases, whereas non-HFO IEDs show only mild pre-peak activation followed by post-peak suppression. Moreover, many neurons preferentially fire during HFO-associated events, suggesting the existence of subtype-specific epileptic microcircuits.^9^ Whether the IEDs analysed by Whitmarsh *et al*.^2^ were associated with HFOs therefore remains an open question and may represent an important source of physiological variability.

Several important gaps persist in the multiscale interpretation of IED correlates. Understanding neuronal dynamics across microcircuits and distributed networks requires consideration of the underlying cellular populations whenever possible. In a human in vitro model, physiological synchronous events rely on a finely balanced interaction between excitatory and inhibitory neuronal activity, whereas epileptiform synchrony reflects a shift toward enhanced excitability dominated by bursting pyramidal neurons.^10^ Interictal-like spikes appear to be initiated by excitatory cells and potentially terminated by inhibitory interneurons. Distinguishing putative excitatory from inhibitory neuronal populations in future human recordings may therefore help clarify whether the increased firing observed during IEDs reflects distinct cellular dynamics across neuronal subtypes.^11^ Exploring the influence of sleep—and its potential stage-specific selectivity on cellular subtypes—would add significant value to clarifying these dynamics.

Another issue concerns the spatial relationship between macroelectrode IEDs and the neuronal populations recorded by microwires. To what extent do recorded single units truly contribute to the generation of the macroelectrode discharge? Although the distance between microwires and macrocontacts may sometimes be minimal, macroelectrode IEDs can also reflect broader field phenomena partially dissociated from locally sampled neurons, particularly across different IED morphologies or HFO-associated events. Direct comparisons between IEDs recorded on microelectrodes and adjacent macroelectrodes remain surprisingly scarce. A more integrated multiscale understanding of IEDs will probably require hybrid SEEG electrodes incorporating microwires or tetrodes between macrocontacts.^8^ Simultaneous neocortical and mesial temporal recordings in the same subjects could help bridge single-neuron activity, LFPs, and large-scale network dynamics across behavioural and sleep states.

Finally, it is difficult to discuss sleep-related IED dynamics without considering memory consolidation, as IEDs may compete with physiological consolidation processes.^12^ In humans, replay of learned neuronal sequences has been shown during sleep, and recent mesial temporal recordings revealed coordinated hippocampo-cortical information flow during ripple–slow oscillation–spindle coupling.^13^ During wakefulness, the degree to which single-neuron activity is modulated by hippocampal IEDs predicts declarative memory disruption.^14^ In parallel, during slow wave sleep, IED–spindle coupling alters the spatial propagation of sleep spindles across cortical networks, suggesting a mechanism by which local epileptic events may induce widespread network dysfunction independently of seizures themselves.^15^ Yet despite these converging observations, the neuronal dynamics linking IEDs, sleep oscillations, and memory consolidation remain largely unexplored.

Although Whitmarsh *et al*.^2^ demonstrate decreased firing rates during the post-IED slow wave at the local microcircuit level, it remains unknown whether comparable inhibitory phenomena occur in distant structures—as previously suggested for fast ripples (HFOs)^8^—or whether such modulation varies across sleep stages.

The unresolved question of REM sleep remains equally fascinating. While NREM sleep clearly facilitates interictal synchronisation, REM sleep often suppresses epileptiform activity despite maintaining sustained neuronal activation.^1^ Understanding why pathological synchronisation collapses during REM, and comparing these dynamics with wakefulness, may provide important clues regarding both seizure generation and physiological brain-state regulation.

Addressing these challenges will require larger multicentric datasets combining microelectrode recordings across multiple anatomical targets together with methodological advances to process such multiscale data. Such efforts are increasingly feasible with the growing use of hybrid SEEG electrodes across centres worldwide, including combined mesiotemporal and extratemporal recordings within the same patients. Future studies may reveal that neuronal responses to IEDs are shaped not only by vigilance state but also by the intrinsic physiology of the underlying anatomical substrate. The study by Whitmarsh *et al*.^2^ provides a valuable illustration of this effort.

Understanding IEDs therefore requires the integration of multiple spatial and temporal scales, from single neurons to large-scale oscillatory networks, and from transient spikes to circadian organisation. More broadly, epileptic discharges should be viewed not as isolated electrical events, but as dynamic neuronal phenomena embedded within the physiological architecture of the sleeping brain.
